# Direct Splash Dispersal Prevails over Indirect and Subsequent Spread during Rains in *Colletotrichum gloeosporioides* Infecting Yams

**DOI:** 10.1371/journal.pone.0115757

**Published:** 2014-12-22

**Authors:** Laurent Penet, Sébastien Guyader, Dalila Pétro, Michèle Salles, François Bussière

**Affiliations:** INRA, UR1321, ASTRO Agrosystèmes tropicaux, F-97170, Petit-Bourg (Guadeloupe), France,; Portland State University, United States of America

## Abstract

Plant pathogens have evolved many dispersal mechanisms, using biotic or abiotic vectors or a combination of the two. Rain splash dispersal is known from a variety of fungi, and can be an efficient driver of crop epidemics, with infectious strains propagating rapidly among often genetically homogenous neighboring plants. Splashing is nevertheless a local dispersal process and spores taking the droplet ride seldom move farther than a few decimeters. In this study, we assessed rain splash dispersal of conidia of the yam anthracnose agent, *Colletotrichum gloeosporioides*, in an experimental setting using a rain simulator, with emphasis on the impact of soil contamination (i.e., effect of re-splashing events). Spores dispersed up to 50 cm from yam leaf inoculum sources, though with an exponential decrease with increasing distance. While few spores were dispersed via re-splash from spore-contaminated soil, the proportion deposited via this mechanism increased with increasing distance from the initial source. We found no soil contamination carryover from previous rains, suggesting that contamination via re-splashing from contaminated soils mainly occurred within single rains. We conclude that most dispersal occurs from direct splashing, with a weaker contribution of indirect dispersal via re-splash.

## Introduction

Aerial dispersal of microbes and fungal spores was described in the late nineteenth century [1] and subsequently developed into its own branch of biology (aerobiology; [2],[3]). The impact of climate on aerial dispersal was soon recognized, especially factors involving mist, fog and rain [4], and wind [5]. Fungal species differ greatly in their ability to disperse through the air [6], and these differences may be related to their dispersal structures that impact the different steps of dispersal: takeoff, distance traveled and landing [7]. Two major types of rain dispersal can be distinguished: “dry dispersal” (i.e. drops dislodge spores at impact, but do not hydrate them; [8]), and “wet dispersal” with spores hydrated before they are dispersed by raindrops or splashes on plants or the ground ([9],[10]). “Dry” dispersal generally occurs as a single dispersal event, while “wet” dispersal may occur recurrently via re-splashes ([1],[11]). Wind will increase primary rain dispersal distance in a downwind direction and decrease it upwind, but it is generally accepted that wind dispersal distances are generally longer than rain dispersal alone [12], and that pure splash dispersal is mostly local [13]. Despite this limitation, splash dispersal may still be particularly efficient when host plant species occurs at high densities, and it is thus especially well-suited for crop pathogens with large patches of host plants growing in often pure, high-density stands.

Since dispersal is a key process in pathogen life cycles, distance traveled from source is one of the main objects of epidemiological studies (e.g., [14]). Even if rain-splash dispersal is a rather local process, it is important to understand how rain characteristics influence the distance traveled by spores via splashing. Indeed, splash dispersal may vary with of rain intensity, duration and frequency, size and velocity of falling drops, wash-off of spores just dispersed. Usually rain intensity does not increase dispersal rate per se ([9],[15]), but it may more specifically enhance spore release and thus the total number of spore dispersed and the probability of long distance dispersal events [7]. On the other hand, more intense rain may decrease effective dispersal by washing off spores just dispersed [15], so that intensive rains may not always translate into increased rates of pathogen dispersal. In contrast, drop size may be more important than rain intensity, with bigger drops being more efficient in spore displacement by splash [1]. In still air, splashes can disperse spores up to about 70 cm [1], and models demonstrate that the detailed mechanism of water splashing is best described as a function of inertia between impacting drops and surface tension at the point of impact [16]. Interface interactions between chemical and physical properties of spores and water will also probably influence dispersal rate [16], but little is known specifically about this at a biological level.

Environmental factors other than rain, splash and spore characteristics will increase or impede dispersal locally: for example, soil nature, soil cover, plant canopy and host density all have strong effects on spore dispersal by splashes. First, vegetation may act as a barrier, putting neighbor hosts beyond reach. Indeed, ground cover was demonstrated as an efficient way to limit splash dispersal, with neighboring canopy leaves reducing dispersal by 90% compared to naked ground [12] and presence of weeds is estimated to decrease overall dispersal distance by half [17]. Splashing dispersal also varies within canopy stages, with dispersal from the upper sides of leaves causing infection at the tips of leaves while splashes from the lower side causing infection at the base of leaves [18]. Moreover, splashing effects vary within and among species ([19],[20]). Secondly, soil topography and composition affect splash dispersal greatly: for example, mulch or straw cover reduces dispersal more than plastic sheet cover ([12],[21]), even on sloping ground (though recurrent lateral and backward splashes can still move spores uphill, e.g. [22]). Dispersal may further differ between soils of varying wetness, as spores dispersed from focal source are more numerous in inundated soils than in bare soils [17]. Surprisingly, few studies investigated the potential effect of prior rains and ground contamination in splash dispersal. It is known that spores present in muddy soils can be splashed further during rains as is already documented in natural settings [23], but little is known about the effect of prior soil contamination on splash dispersal.

In this study, we assessed rain splash dispersal of *Colletotrichum gloeosporioides* (Penz.) Penz. and Sacc conidia in an experimental setting using a rain simulator (as suggested by Reynolds [24]); with emphasis on re-splash of spores from prior soil contamination. We asked the following questions: what is the relative importance of initial direct splashing and re-splashing in local pathogen dispersal? Can spores from prior ground contamination be dispersed by a subsequent rain?

## Material and Methods

### Material and spore production

*Colletotrichum gloeosporioides* (Penz.) Penz. and Sacc is a species complex of fungi that are mostly plant pathogens ([25], though they are also reported as harmless endophytes in a few cases, see [26]), and these fungi attack many crop species worldwide [27]. In the Greater Yam (*Dioscorea alata* L.), *C. gloeosporioides* causes anthracnose disease, resulting in leaf decay during infection. Conidia are dispersed locally by rains (rain splash dispersal; [27]) and infect further leaves of neighboring plants, sometimes leading to severe epidemics resulting in important economic losses [28] in a crop already subject to high variation in yield [29]. In this experiment, we used *C. gloeosporioides* strain #220 from our lab (strain not deposited in international repository but available on request). This strain is of Guadeloupean origin; it was isolated in 1999 on the *D. alata* variety Kabusah and demonstrates moderate virulence on most common local yam varieties. This strain bears a diagnostic PCR fragment of *C. gloeosporioides*, amplified using the primers ITS4 [30] and CgInt2 [31]. For spore multiplication, spores were first grown on a DPDA media and then conidia were diluted in water and sprayed onto cut leaves of the highly susceptible yam *D. alata* var. Plimbite. Infected leaves were placed in a water sprayed plastic bag and left for incubation for four days at room temperature (ca 25°C), until leaves had the characteristics of heavy anthracnose infection (numerous spread necroses).

### Dispersal experiment

**Figure 1:**
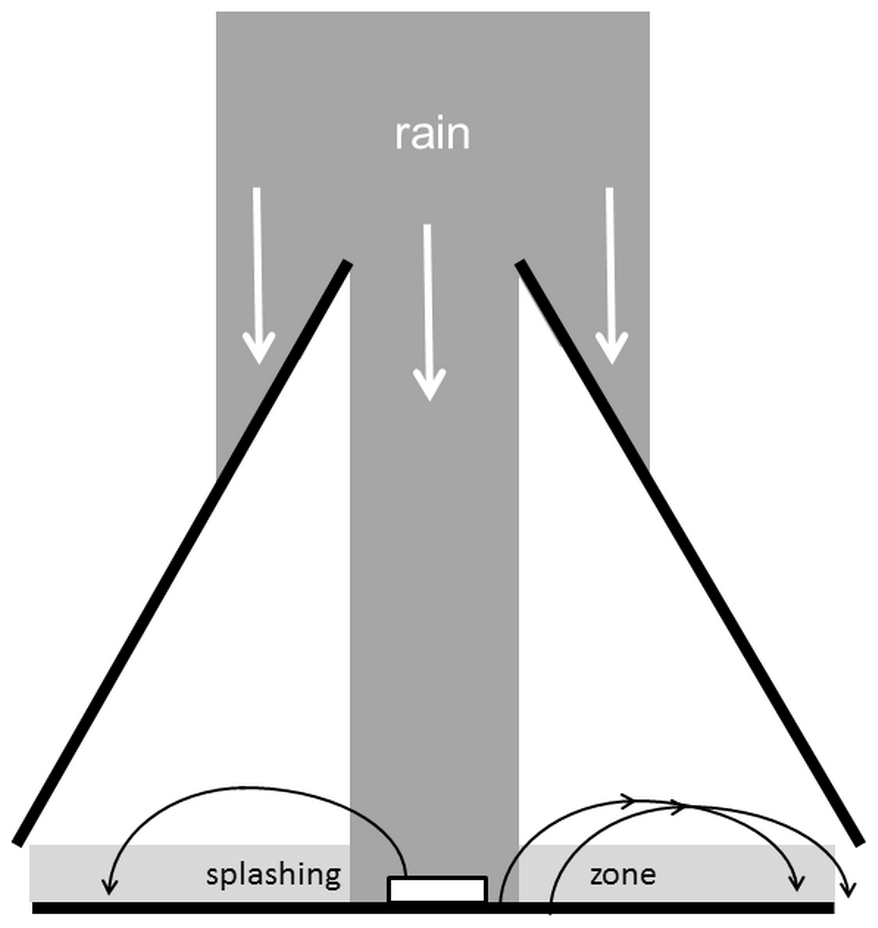
Experimental set-up for artificial rain. Artificial rain was canalized by a conic tent with an open of 30 cm in diameter. Inoculum sources (Petri dishes with Cotton Blue solution and spores) were placed at the center of the rain zone. Microscope slides were placed in concentric circles around each source (at distances from 24 to 45 cm), and received spores either directly from rain splash from source (blue drops spores) or indirectly via re-splashes from ground (clear drops spores).

We investigated rain splash efficiency with experimental rains following Reynolds et al. [24]. Artificial simulated rain was produced for two minutes for each assay (run) with a Deltalab Microprocessor Controlled Spray System, EID 330, developed by IRD (Asseline & Valentin, 1978) used to simulate rain in still air. Rainfall was simulated by a constant speed oscillating nozzle (Deltalab, Tec Jet SS 6560) with a sweep angle of 120° positioned at a height of 2.50 m. Water pressure at the nozzle orifice was 62 kPa. For inoculum sources we used infected yam leaf fragments with sporulating necroses placed in Petri dishes with 10 mL distilled water and Cotton Blue five minutes prior to runs in order to stain spores. These petri dishes were placed at the center of the rain assay in a conic tent with a 30 cm diameter opening at the top (see Fig. 1). The ground surface in the tent was covered in rough paper sheets (plain “Joseph” paper grade 551, purchased from VWR, reference 111-500x) that were changed every two runs, thus allowing for a non-contaminated soil treatment (odd number runs) and soils with a first rain contamination (even number runs). For each run, 60 microscope slides (plain microscope slides, W×D×H: 76×26×1 mm, purchased from VWR ref. 631–1552) were placed in concentric circles around the spore source at specific distances (24, 31, 38 and 45 cm from source). Experimental rain led to the dilution of the inoculum sources because about 8 mL of rain water on average fell into the petri dishes. Nonetheless they were still heavily blue and ‘source spores’ were still strongly stained, so that we could differentiate between spores directly dispersed (i.e. blue stained spores), and spores indirectly dispersed via re-splash (rinsed, colourless spores) during counting.

### Spore counts

We estimated spore concentrations in the inoculum source before (i.e., initial spore solution) and after rain treatment (i.e., residual spore solution, accounting for dilution) with sampling and counting spores on a hemacytometer under a light transmitted microscope. Initial spore solution was not controlled for variation in spore concentration but we used concentration as covariate in analyses (initial spore concentration), and the range for the twelve runs was 2.68–5.92 ×10^5^ spores.mL^−1^ (mean concentration: 4.06 ×10^5^ spores.mL^−1^). The total number of exported spores was estimated for each rain (hereafter run), and ground contamination was estimated as exported spores left between runs two runs. We estimated the level of ground contamination as number of spores on the ground left after the rain, i.e., initial minus residual spore estimates from the inoculum source, corrected for dilution due to rain (the difference in spore number before and after rain is the number of spores dispersed within the run, which is also a proxy for contaminating spores left on the ground after the rain). Estimated exported spores (and soil contamination) range were 1.62–4.95 ×10^5^ spores.mL^−1^. Slides were randomly selected for each run and stratified for each sampled distance (only two slides at the 24 cm distance were counted due to heavy contamination loads, 4 slides were counted for all other distances from source). Spores were counted under light transmission microscopy, with whole slide counts. It was easy to distinguish individual drops on slides from all dispersal distances, so we counted drops and spores per drop, distinguishing between blue drops containing blue stained spores (directly dispersed from the Cotton Blue stained petri dish), and pale or colourless spores from rinsed drops (dispersal via re-splashing from ground). We counted 174 slides from 12 runs (6 without prior soil contamination, 6 with contaminated soil).

### Statistical analyses

To avoid pseudo-replication issue for slides within runs, we directly worked on means at the run level. We first conducted a two-way full factorial Anova in R [32], to test whether the number of drops dispersed to slides changed with distance from the petri dish spore source and whether this change differed between directly dispersed blue drops and rinsed, secondarily dispersed colorless drops. Second, we ran an Ancova to test the effect of distance and type of dispersal (primary or secondary, assessed from the color of drops) on mean spore number per drop, with initial spore concentration and previous soil contamination as covariates. We added interaction terms between color of drops and distance to account for the potential impact of inoculum source on direct dispersal (blue drops) and of prior soil contamination on indirect dispersal (washed drops). Third, we conducted an analysis of covariance to test the impact of distance and source of dispersal (color of drops) on total spores dispersed, with initial spore concentration, previous soil contamination as covariates. We added interaction terms between color of drops and distance to account for the potential impact of inoculum source on direct dispersal (blue drops) and of prior soil contamination on indirect dispersal (washed drops) and differential impact of spores within drops to dispersal. The assumptions of homoscedasticity and normality of residuals were met in all analyses.

### Path analyses

We used path analysis modeling [33] to examine the effect of distance on direct and indirect dispersal and how the different modes of dispersal (primary and secondary) influence total number of spores dispersed (S1 Table). Path analysis is a regression method that tests an *a priori* predetermined subsample of all possible correlations between covariates in order to focus on specific hypotheses of direct and indirect dependence between covariates. We conducted these analyses using PROC REG and PROC CALIS (SAS Institute Inc., Cary, NC, USA), using structural equation modeling, covariance matrices, and estimating path gradients with maximum likelihood. Prior to the analysis, data were checked and extreme outliers identified with Cook's D values [34]. Eight data points, all >10,000, were identified as exceedingly deviant from the rest of the data and were discarded from further analyses. Independent variables followed leptokurtic L-shaped curves, and we therefore used Box Cox transformations. We used the AID package [35] in R [32] to determine the best exponent for each variable [36], which were λ  =  0.17, 0.3 and 0.38 for number of blue drops, mean spore number per blue drop and mean spore number per rinsed drop, respectively. We first investigated a full model with three exogenous variables (distance, exported spores and prior soil contamination) and four endogenous variables (number of blue drops, mean spore number per blue drop, number of washed drops and mean spore number per washed drop). We investigated the effect of distance and the effect of exported spores on all subsequent endogenous covariates, in addition to the effect of prior soil contamination on number of rinsed drops and mean spore number per rinsed drop. The initial model also included effects of drop numbers on mean spore number per drop (blue and washed respectively) and the further effects of these four covariates on total spores dispersed. Number of rinsed drops was significantly correlated to error term in mean spore number per rinsed drop, and we therefore dropped this covariate from the model to avoid any issue with multi-collinearity [37] and dependencies among covariances. We then further dropped paths with non-significant impact on total spores dispersed and covariates without significant effects (exported spores and prior soil contamination) to generate a minimal model with correctly fitting covariance matrices (model generation strategy, [38]), to the exception of mean spore number per rinsed drop.

Several fit indices were assessed once the best model was identified, and given the moderate sample size (N  =  166), we followed Hu and Bentler's [39] suggestion to favor SMRSR over RSMEA among indices of model fit, because RMSEA makes many type-one errors, rejecting true population models. We indicated diverse fit indices following Hooper recommendations for path reports [40]. The retained model of path had a Chi-squared value of 3.35 (2df, *P*  =  0.187), indicating that the model covariance matrix did not significantly differ from the data covariance matrix, even though the “distance” factor deviated from a normal distribution. As reported in the SEM and Path literature (see [40]), Chi-squared may suffer from lack of power and be inadequate because of our moderate sample size. We followed Hooper's suggested fit index report: the RSMEA estimate is 0.064 (95% CI  =  0–0.18), which is slightly above the recommended conservative rejection threshold for fair fit. Given the ML method used here and moderate sample size (N  = 166), we followed Hu & Bentler's suggestion to use SMRSR instead [36]. SMRSR for the retained model is good (0.025) and Bentler Comparative Fit Index very good (0.995).

## Results

We found much more dispersal via direct dispersal from source, with re-splashing contributing only weakly to local dispersal, though with an increasing contribution at greater distances, thus increasing the probability of infection of neighboring plants in fields. Prior ground contamination had nevertheless no significant impact on further dispersal by rains suggesting no carryover of spores between rainfalls.

### Effect of splashing and re-splashing

**Figure 2:**
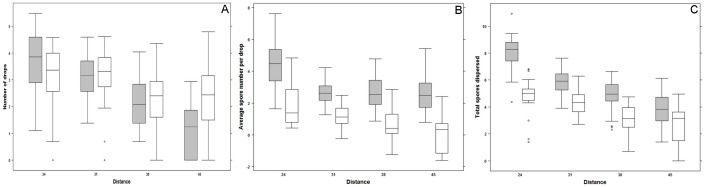
Comparison of direct and indirect splashing efficiency as a function of distance. A. Total drop numbers, B. Mean Spore Number per Drop, and C. Total Number of Spores dispersed. Scales on Y-axes are logarithmic for each plot. Grey boxes stand for direct splashing from source (blue drops) and white boxes stand for indirect re-splashing events (clear drops). Data points outside 95% confidence interval are symbolized with circles.

Drop number decreased with distance from source (Table 1A; Fig. 2A), and as expected this effect was more pronounced for direct splashing from the inoculum source (blue drops) than for re-splash (rinsed drops; significant interaction between distance and drop color, see Table 1A). Re-splash drops are pushed forward sequentially during the rain. Indeed, drop distribution is different between short distances –i.e. 24 and 31 cm (with higher number of drops since it belongs to the filtering zone and received drops both from rain and re-splashes), and greater distances –i.e. 38 and 45 cm (where drop numbers were lower, see Fig. 2A). As a consequence, while direct splashes from inoculum source had a great contribution to total number of drops at short distance, re-splash drop number contributed more to the total number of drops at the distance of 45 cm. Despite this greater contribution of re-splash at long distance, average spore numbers per drop was always greater in blue drops, and the number of spores dispersed indirectly via rinsed drops declined with distance while it held relatively constant in blue drops (Fig. 2B, significant interaction between distance and drop color, see Table 1B). Overall, most spore deposition was via direct splashes (blue drops), even if the difference between direct and indirect dispersal decreased with distance (Fig. 2C). Both direct splashing and indirect re-splashing contributed to dispersal but there was also a significant interaction between distance and drop color (blue drops always decreasing with distance while washed drops were plateauing; Fig. 2A, Table 1A). The interaction between distance and drop color was also highly significant (mean spore number per rinsed drops always decreasing with distance while mean spore number per blue drops were plateauing after 24cm; Fig. 2B, Table 1B).

**Table 1:** Effect of distance, source of dispersal (color of drops: blue for direct dispersal and rinsed for indirect dispersal) and their interaction on dispersal parameters: A) Number of Drops, B) Mean Spore Number per Drop and C) Total Spores Dispersed. Significance is emphasized by P-values in bold.

A.				
	Df	Mean Square	F	P
Distance	1	26966	46.799	**<0.0001**
Color	1	756	1.312	0.26
Distance x Color	1	7843	13.611	**0.0004**
Residuals	92	576		

B				
	Df	Mean Square	F	P
Distance	1	140748	9.481	**0.0027**
Color	1	117991	7.948	**0.0059**
Initial Spore Solution	1	56158	3.783	0.0549
Prior Soil Contamination	1	14289	0.962	0.33
Distance x Color	1	114457	7.710	**0.0067**
Residuals	90	14846		

C.				
	Df	Mean Square	F	P
Distance	1	90527221	26.888	**<0.0001**
Color	1	53118135	15.777	**<0.0001**
Initial Spore Solution	1	7802367	2.317	0.77
Prior Soil Contamination	1	4560188	1.354	0.25
Distance x Color	1	77367051	22.98	**<0.0001**
Residuals	90	303009049		

### Spore dispersal and prior soil contamination

Mean spore number per drop and total spore dispersed were significantly affected by distance (decreasing dispersal, see Fig. 2B and 2C), by source of dispersal (drop color) but were not impacted by initial spore solution concentration nor by levels of prior soil contamination (see Table 1B and 1C). This suggests that re-splashes are probably less sensitive to the number of spores available to dispersal in either leaf inoculum or contaminated ground and maybe more sensitive to physical properties of rain and drop (not evaluated in the present study). Therefore, spores previously dispersed are not contributing significantly to dispersal with further rains. Contamination mostly occurs via direct splashing or re-splashes within single rain events.

### Path analysis

**Figure 3:**
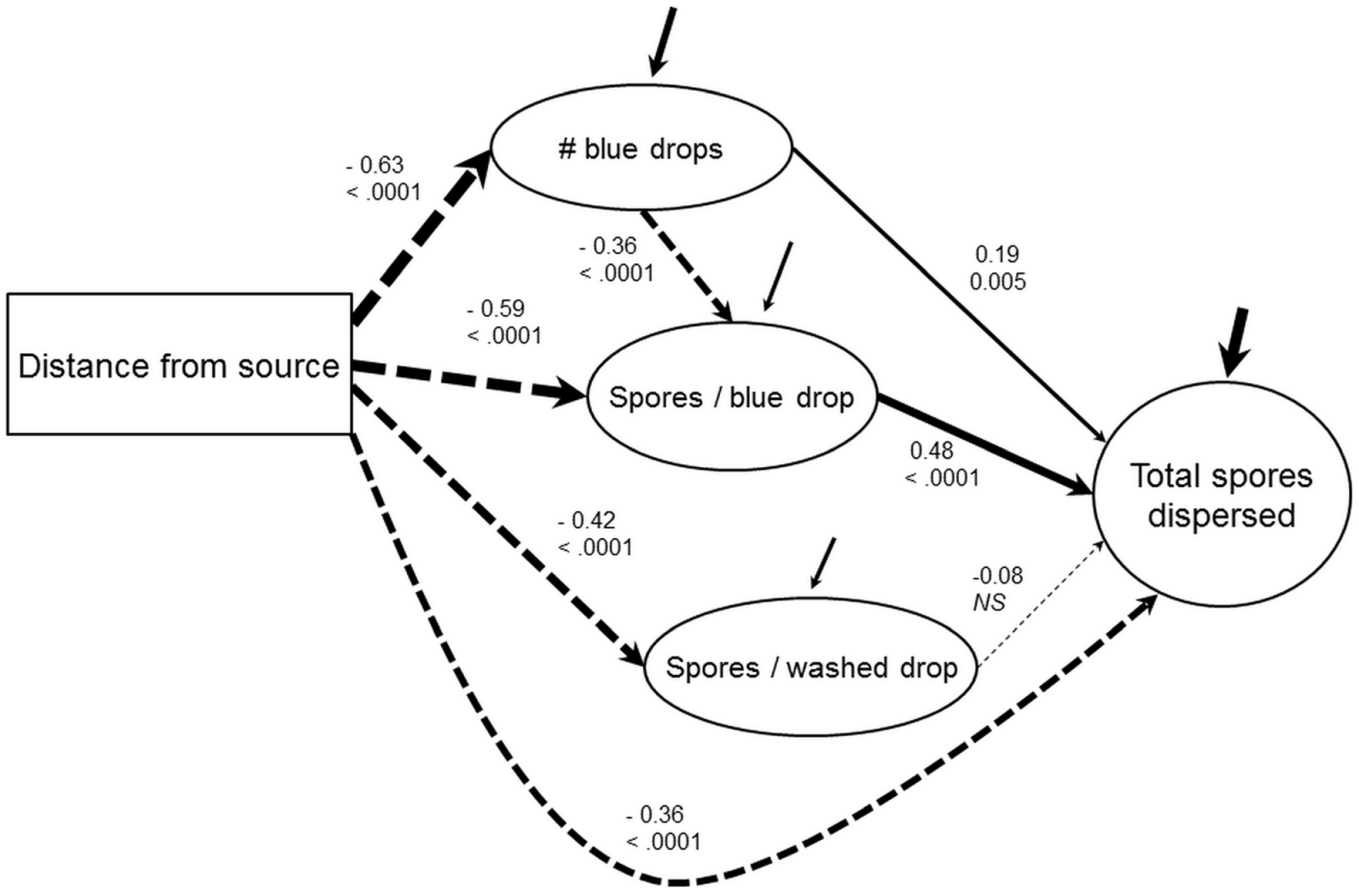
Path diagram of spore dispersal via splashing in the experimental setting. Direct effects are represented. Dash lines stand for negative path coefficients, solid lines for positive paths coefficients. Significance of path gradients are indicated below the standardized regression value (NS  =  non-significant, *P* value otherwise). Path sizes are drawn proportional to standardized value of gradients (including unexplained variance for endogenous variables, incoming arrows).

Distance, number of blue drops and mean spore number per blue drops had highly significant impact on dispersal (Fig. 3), but mean spores per rinsed drop had a weaker and insignificant impact in our path model, possibly because of its interaction with distance. All components of spore dispersal, but especially the measure of direct dispersal (number of blue drops) decreased with increasing distance from the spore source. Mean spore number per blue drop had the strongest impact on dispersal and was more important than number of blue drops (path gradients of 0.48 vs. 0.19 respectively) and was thus the major driver of spore dispersal. Interestingly, number of blue drops was negatively correlated to mean spore number per blue drop (-0.36), demonstrating that increased opportunities for direct dispersal did not necessarily translate into a global increase in spore dispersed. Indeed, incident drops may have broken up into several smaller droplets, each containing fewer spores, though this was not investigated in the present study.

## Discussion

We found that splash dispersal is a local process occurring mainly at small distances, and most importantly direct dispersal dominates indirect re-splash, the latter contributing only in a limited fashion in our experiment (Fig. 3). Previous ground contamination contributed only little to dispersal via re-splashing, so dispersal mostly occurs within single rains. Direct dispersal from a spore source had the greatest effect because more spores were dispersed in single drops (Fig. 2B), even if indirect dispersal via re-splash contributed relatively more drops at greater distance (Fig. 2A). Indeed, only a few spores were displaced via re-splashing, resulting in fewer spores dispersed (Fig. 2C). In the following discussion, we will relate these findings to their impact on dispersal by rains, both in the context of *Colletotrichum gloeosporioides* infecting yams and in a more general context of splash dispersal.

### Rain effects on spore splash dispersal

Rain splash is known to take place at very small scales [11], and results in smaller dispersal distance than wind ([6],[41]). Our experiment did not investigate the full range of splashing dispersal, but our results confirm that a majority of spores dispersed by rain are dispersed at short distance from the inoculum. Since direct dispersal is heavily dependent on chance events, one would expect re-splashing events, which allow for even further propagation of released spores, to eventually become of a greater importance for dispersal than direct splashes. Our design allowed us to compare these two components of dispersal (direct splash and indirect via re-splashes), and indirect dispersal does not exceed direct dispersal because direct splashes carried a dramatically greater number of spores per drop compared to re-splashed drops (figure 2B). This is the first study comparing the efficiency of direct versus indirect dispersal, so we cannot know if other species behave similarly. Species of *Colletotrichum* differ in direct splash dispersal efficiency as a function of distance, probably due to differences in mucilage matrix properties [42]. We may also expect differential ability to disperse via re-splash, for example if variation in spore size or shape has an impact on dispersal capacity, or if differences in cell wall hydrophilic properties affect adherence to forming splashes. In the current context of yam cropping, ridge interspacing falls within the direct dispersal distance. Ridges may therefore intercept direct splashes and also decrease re-splash efficiency, while staking is further increasing odds of direct drops intercept. Both agronomic practices are rooting in productivity management (sparing space and concentrating production), but they also ease direct dispersal of *Colletotrichum gloeosporioides*. The pressure to evolve re splash dispersal efficiency is thus lowered. It would be interesting to test whether re-splash dispersal ability is varying across pathogens species in different cropping systems.

### Prior soil contamination effects on spore dispersal by rains

Soil characteristics can impact splashing dispersal in two ways, first during indirect dispersal via re-splash during rains, and second via indirect dispersal in subsequent rain events due to prior contamination. While our experimental ground surface was a rough paper sheet and would not realistically compare to natural soils, it would probably behave more as a dispersal enhancer than natural soils because its clearance ability (drainage) is low. Indeed, the experimental ground became saturated and could not absorb water very rapidly after rains begin, and quickly formed a water film. Re-splash within rains could have thus been facilitated by the low clearance and rapid film formation. The effect of re-splash in the experiment was low compared to direct dispersal from an inoculum source, but success in dispersal only requires a single spore to contaminate a new host, so that indirect dispersal may still provide a way to establish in new hosts. While of secondary importance compared to direct splashing, re-splashing may indeed still be a major source of spore contamination in natural crop conditions. It would be important to investigate the effect of different soil characteristics on pathogen dispersal compared to other factors such as plant cover that is known to influence splash dispersal (barrier effect, [43]). Indeed, little is known as to how soil characteristics influence splash dispersal of spores (but see [44]). Because little is known about soil contributions to rain dispersal of pathogens, further experiments should address the diversity of soil clearance ability and microtopography and their consequences on dispersal.

Secondly, prior soil contamination had no detectable effect on spore dispersal in our experiment (Table 1), i.e. the presence of spores dispersed during previous rain did not detectibly increase spore loads in the following rain event. Perhaps spore density on the ground surface was too low to have an impact via re-splash or perhaps deposited spores were trapped on the surface. This could happen under natural conditions particularly if spores become bound to humus compounds. The paper sheets we used were highly fibrous and might compare to humus-rich or mulched soils with regard to such trapping or retention effects. However, if other soil types have different soil retention capacities, our lack of resplash effects could be an artifact of the paper used, and may not generalize to most crop conditions. It is also possible that the retention effect is strong regardless the nature of the soil and few spores can be remobilized in further rains. Several studies emphasize the importance of takeoff but there might be a cost to landing/deposition [7] whenever spores are not deposited on a suitable host, resulting in major spore loss after direct dispersal especially losses precluding indirect further dispersal by re-splash and potential indirect dispersal via re-splash. *Colletotrichum gloeosporioides* is known to have a very low survival in soils, even when settling in decaying plant pieces, though they may survive longer on plant residues [45] (long survival time has nevertheless already been reported, see [46]). We would thus expect dispersal to be successful only if dispersed spores quickly land on a new host. The inability of this pathogen to survive outside its host is probably a key factor determining the apparent weak impact of soil contamination on further splash dispersal.

In summary, we demonstrated that direct dispersal from a source is the main mode of spore dispersal by rain-splashes and that indirect dispersal via re-splash has a smaller impact but can still contribute somewhat to spore contamination. However, rain splash dispersal is limited to single rain events, and once spores have settled, subsequent rains do not seem to promote subsequent dispersal: prior soil contamination was not an important factor in spore dispersal in *Colletotrichum gloeosporioides*. While both rain and cover characteristics are known to impact rain splash dispersal, little is known about soils characteristics or diversity in pathogen dispersal abilities via splashing, and we suggest this is a gap to fill in further experiments to increase our understanding of rain splash.

## Supporting Information

S1 Table **Experiment dataset.** Distance is in cm, TotNbDrops, NbWhiteDrops, NbBlueDrops, TotNbSpores, NbWhiteSpores, NbBlueSpores, MeanSporeNbAllDrops, MeanSporeNbWhiteDrops, MeanSporeNbBlueDrops are expressed as spore numbers and InitialSporeSolution, RainedSporeSolution, ExportedSpores, PreviousSoilContamination are expressed as 10^5^ spores.
(XLS)
